# Machine learning identifies characteristics molecules of cancer associated fibroblasts significantly correlated with the prognosis, immunotherapy response and immune microenvironment in lung adenocarcinoma

**DOI:** 10.3389/fonc.2022.1059253

**Published:** 2022-11-09

**Authors:** Qian Wang, Xunlang Zhang, Kangming Du, Xinhui Wu, Yexin Zhou, Diang Chen, Lin Zeng

**Affiliations:** ^1^ Hospital of Chengdu University of Traditional Chinese Medicine, Chengdu, Sichuan, China; ^2^ Department of Geriatric, Hospital of Chengdu University of Traditional Chinese Medicine, Chengdu, Sichuan, China; ^3^ Department of Vascular Surgery, Hospital of Chengdu University of Traditional Chinese Medicine, Chengdu, Sichuan, China; ^4^ Guangxi University of Chinese Medicine, Nanning, China; ^5^ Department of Urology Surgery, Hospital of Chengdu University of Traditional Chinese Medicine, Chengdu, Sichuan, China; ^6^ Department of Neurosurgery, Hospital of Chengdu University of Traditional Chinese Medicine, Chengdu, Sichuan, China

**Keywords:** cancer-associated fibroblasts, lung adenocarcinoma, immunotherapy, TIDE, immune microenvironment

## Abstract

**Background:**

Lung adenocarcinoma (LUAD) is a highly lethal disease with a dramatic pro-fibrocytic response. Cancer-associated fibroblasts (CAFs) have been reported to play a key role in lung adenocarcinoma.

**Methods:**

Marker genes of CAFs were obtained from the Cell Marker website. Single sample gene set enrichment analysis (ssGSEA) was used for CAFs quantification. R and GraphPad Prism software were utilized for all analysis. Quantitative real-time PCR (qRT-PCR) was utilized to detect the RNA level of specific molecules.

**Results:**

Based on the ssGSEA algorithm and obtained CAFs markers, the LUAD patients with low- and high-CAFs infiltration were successfully identified, which had different response patterns to immunotherapy. Through the machine learning algorithm – LASSO logistic regression, we identified 44 characteristic molecules of CAFs. Furthermore, a prognosis signature consisting of seven characteristic genes was established, which showed great prognosis prediction ability. Additionally, we found that patients in the low-risk group might have better outcomes when receiving immunotherapy of PD-1, but not CTLA4. Also, the biological enrichment analysis revealed that immune response-related pathways were significantly associated with CAFs infiltration. Meanwhile, we investigated the underlying biological and microenvironment difference in patients with high- and low-risk groups. Finally, we identified that AMPD1 might be a novel target for LUAD immunotherapy. Patients with a high level of AMPD1 were correlated with worse responses to immunotherapy. Moreover, immunohistochemistry showed that the protein level of AMPD1 was higher in lung cancer. Results of qRT-PCR demonstrated that AMPD1 was upregulated in A549 cells compared with BEAS-2B. Meanwhile, we found that the knockdown of AMPD4 can significantly reduce the expression of CTLA4 and PDCD1, but not CD274 and PDCD1LG2.

**Conclusion:**

We comprehensively explored the role of CAFs and its characteristics molecules in LUAD immunotherapy and developed an effective signature to indicate patients prognosis and immunotherapy response. Moreover, AMPD1 was identified as a novel target for lung cancer immunotherapy.

## Introduction

Lung cancer remains a threatening disease globally, despite declining incidences and mortality rates (1). The majority of lung cancer patients suffer from lung adenocarcinoma (LUAD). Generally, the development of LUAD is influenced by air pollution, smoking, and other factors (2). Most patients with LUAD remain incurable despite advances in treatment over the past decade (3). For this reason, the poor prognosis of LUAD patients demands a more mature early diagnosis and treatment strategy. Nowadays, combining chemoradiotherapy with immunotherapy has become the most common treatment for patients who cannot undergo surgery or resection of their cancers.

Cancer-associated fibroblasts (CAFs) is a type of activated fibroblast that is associated with cancerous cells. A significant role of CAFs is believed to be played in cancer progression (4). Multi-faceted responses by CAFs to stress in tumor environments allow them not only to adapt to tough conditions but also to enhance pro-tumorigenic behavior (5). CAFs are major tumor-promoting components in the tumor microenvironment (TME) of LUAD. According to one study, miR-210 was upregulated in CAFs-derived exosomes, enhancing cell migration, proliferation, invasiveness, and epithelial-mesenchymal transition (EMT) in lung cancer cells (6). To develop novel LUAD treatments and technologies targeted at CAFs, additional studies are needed to elucidate how tumor parenchyma and stroma interact during tumorigenesis (7).

The immune system monitors and destroys cancer cells, but tumor cells can circumvent this natural defense and develop tolerance (8). The immune system normally protects healthy tissue from cytotoxic immune reactions triggered by infection, but tumor cells can use the same immune checkpoints to evade immune destruction (9). By recognizing tumor antigens, T cells release interferon-x (INF-x), which attracts other cytotoxic immune cells and activates checkpoints. The use of immunotherapy has been beneficial to many LUAD patients, and the expression of PD-L1 helps identify those patients who are likely to respond (10). Consequently, exploring the biomarkers for predicting immune responses to lung cancer is important to maximize the effectiveness of new immunotherapy drugs (11).

High-throughput molecular technologies can help us identify genomic biomarkers that can be used to tailor precision medicine to individual patients using genomic biomarkers (12). In addition to being powerful diagnostic tools, these biomarkers also provide excellent prognostic information. The development of immunotherapy in LUAD treatment has shown exciting potential in recent years. Therefore, it is important to conduct prospective research on the biological molecules and mechanisms that affect LUAD immunotherapy.

## Methods

### Datasets downloaded

In addition to transcriptome expression data, The Cancer Genome Atlas (TCGA) and Gene Expression Omnibus (GEO) databases also provide clinical information of LUAD patients. For the TCGA database, the TCGA-LUAD project was selected and the initial expression profile file was “bcr-xml”. Data in GSE68465 was downloaded from the GEO database in MINiML format, which contains the corresponding platform file GPL96 (13). Data were normalized by “preprocessCore” package in R software. Annotation information of the platform is used to convert probe IDs into gene symbols. For a gene with multiple probe IDs, the mean value of these probe values was identified as the gene expression. The baseline feature of patients in TCGA and GSE68465 were shown in **Table 1** and **Table 2**.

**Table 1:** Baseline information of patients in TCGA.

Clinical features		Number	Percentage (%)
Age	<=65	241	46.2
	>65	262	50.2
	Unknown	19	3.6
Gender	Female	280	53.6
	Male	242	46.4
Stage	Stage I	279	53.4
	Stage II	124	23.8
	Stage III	85	16.3
	Stage IV	26	5.0
	Unknown	8	1.5
T-stage	T1	172	33.0
	T2	281	53.8
	T3	47	9.0
	T4	19	3.6
	Unknown	3	0.6
M-stage	M0	353	67.6
	M1	25	4.8
	Unknown	144	27.6
N-stage	N0	335	64.2
	N1	98	18.8
	N2	75	14.4
	N3	2	0.4
	Unknown	12	2.3

**Table 2:** Baseline information of patients in GSE68465.

Clinical features		Number	Percentage (%)
Age	<=65	231	52.1
	>65	212	47.9
Gender	Female	220	49.7
	Male	223	50.3
T-stage	T1	150	33.9
	T2	251	56.7
	T3	28	6.3
	T4	12	2.7
	Unknown	2	0.5
N-stage	N0	299	67.5
	N1	88	19.9
	N2	53	11.9
	Unknown	3	0.7

### Single sample gene set enrichment analysis

The ssGSEA method was used to quantify the individual scores for each tumor sample (14). The ssGSEA algorithm computes overexpression measures for a list of genes of interest using a rank-based method. The list of CAFs markers genes obtained from the Cell Marker website (http://bio-bigdata.hrbmu.edu.cn/CellMarker/) was used to calculate ssGSEA scores.

### Identification of optimal variables

The characteristic gene screening was based on LASSO logistic regression (15). LASSO logistic regression is a popular variable selection method that was implemented using the “glmnet” package.

### Prognosis analysis

Based on the identified characteristics of molecules, a univariate Cox regression analysis was conducted to screen the prognosis-related genes. Next, the LASSO regression algorithm was utilized for dimensionality reduction. Multivariate Cox-regression analysis was responsible for model construction with the formula “Risk score = “Coeff A * Exp A + Coeff B * Exp B + … + Coeff N * Exp N”. Kaplan-Meier (KM) survival and receiver operating characteristic (ROC) curve was utilized to evaluate the performance of prognosis model.

### Immune-related analysis

Immune infiltration cells in LUAD patients were determined using the CIBERSORT, EPIC, MCPCOUNTER, QUANTISEQ, XCELL and TIMER algorithms (16–20). An evaluation of LUAD immunotherapy was conducted through the Tumor Immune Dysfunction and Exclusion (TIDE) website (21).

### Enriched pathways

Gene set variation analysis (GSVA) and GSEA analysis were conducted based on the Hallmark gene set from MSIGBD project (22). For the GSEA analysis, the normalized enrichment score (NES) of the individual pathway was calculated. The reference gene sets were Hallmark and Gene Ontology (GO).

### Single-cell analysis

A brief single-cell analysis was performed based on the Tumor Immune Single-cell Hub (TISCH) website (23).

### Immunohistochemistry

The immunohistochemistry image of AMPD1 in lung cancer and normal tissue were downloaded from The Human Protein Atlas (HPA) project (https://www.proteinatlas.org/).

### Cell culture

The normal lung cell BEAS-2B and lung cancer cell A549 were previously restored in our laboratory. These cell lines were grown with 1640 medium containing 10% fetal bovine serum.

### Cell transfection

Control and knockdown plasmids were purchased from synthesized by Shanghai Jima Biotechnology. Cell transfection was conducted according to the standard process (24).

### Quantitative-real time PCR

With the help of the Trizol reagent, RNA was isolated from BEAS-2B and A549 cells. Total RNA was extracted with an RNA ultrapure extraction kit, a UV-Vis spectrophotometer was used to determine total RNA concentration and purity (OD260/OD280). The PCR assay was performed with the protocol. The primer sequences are shown below: AMPD1, forward primer, 5′-TATAGTGTCAGTCAGTCACCCC-3′, reverse primer, 5′-GAGTTTGAACAGAGGCATTGTTG -3′; PDCD1, forward primer, 5’-CCAGGATGGTTCTTAGACTCCC-3’, reverse primer, 5’-TTTAGCACGAAGCTCTCCGAT-3’; CTLA4, forward primer, 5’-GCCCTGCACTCTCCTGTTTTT-3’, reverse primer, 5’-GGTTGCCGCACAGACTTCA-3’; PDCD1LG2, forward primer, 5’-ATTGCAGCTTCACCAGATAGC-3’, reverse primer, 5’-AAAGTTGCATTCCAGGGTCAC-3’; CD274, forward primer, 5’- TGGCATTTGCTGAACGCATTT-3’; reverse primer, 5’-TGGCATTTGCTGAACGCATTT-3’; GAPDH, forward primer, 5’-GGAGCGAGATCCCTCCAAAAT-3’, reverse primer, 5’-GGAGCGAGATCCCTCCAAAAT-3’.

### Statistical analysis

R software was used to perform all statistical analyses. Statistical significance was determined by P values under 0.05.

## Result

### CAFs quantification and its role in LUAD

**Figure 1:**
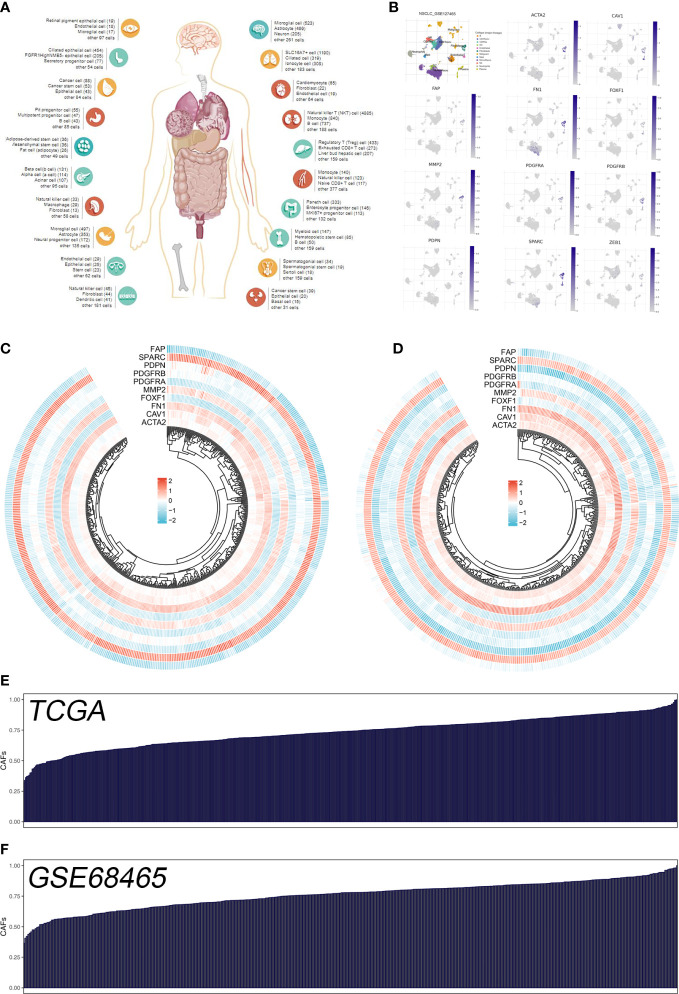
Quantification of the CAFs infiltration based on identified markers. **(A)** The human single-cell atlas from the online website Cell Marker, http://bio-bigdata.hrbmu.edu.cn/CellMarker/; **(B)** The expression map of marker genes in different subgroup cells; **(C)** The heatmap demonstrated the expression of CAFs marker genes in TCGA cohort; **(D)** The heatmap demonstrated the expression of CAFs signature genes in GSE68465 cohort; **(E)** CAFs score in TCGA cohort quantified by ssGSEA; **(F)** CAFs score in GSE68465 cohort quantified by ssGSEA.

**Figure 2:**
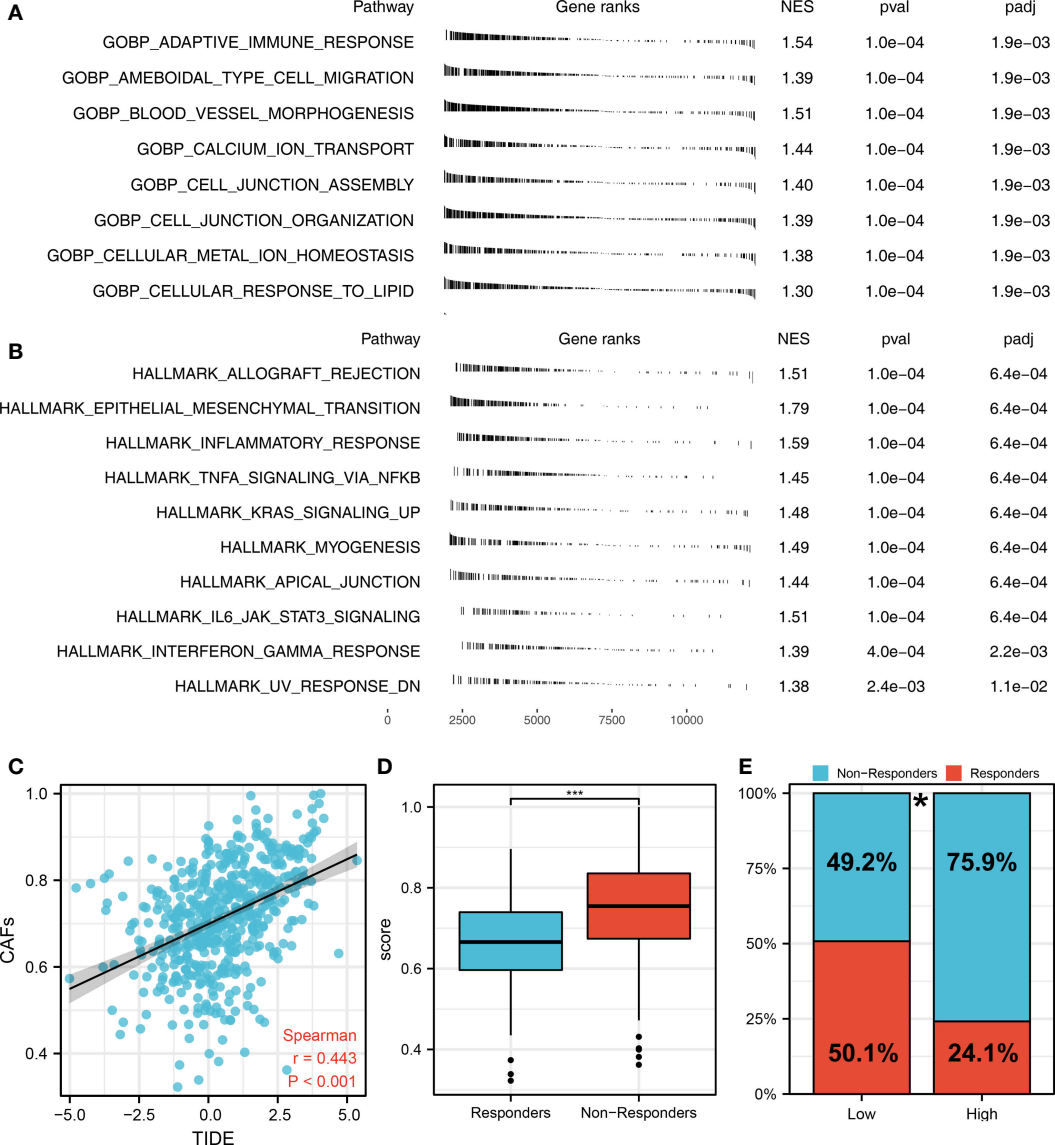
The role of CAFs in affecting lung cancer biological pathways and immunotherapy response. **(A)** The GSEA analysis between high- and low-CAFs groups based on the Hallmark gene set; **(B)** The GSEA analysis between high- and low-CAFs groups based on the GO gene set; **(C)** Correlation analysis between TIDE score and CAFs score; **(D)** CAFs level in immunotherapy responders and non-responders ***P < 0.001; **(E)** Percentage of immunotherapy responders in patients with high and low CAFs infiltration, *P < 0.05.

The flow chart of the whole study was shown in **Figure S1**. The single-cell analysis provides high-dimensional information that allows the identification of CAFs from heterogeneous pools of cells, as well as the ability to cluster transcriptome data into multiple CAFs subtypes based on their signature genes. According to their original annotations, we identified 11 markers of CAFs based on the Cell Marker website (**Figure 1A**). The single cell analysis indicated these markers ACTA2, CAV1, FAP, FN1, FOXF1, MMP2, PDGFRA, PDGFRB, PDPN, SPARC, and ZEB1 were highly expressed in the CAFs of the lung cancer microenvironment (**Figure 1B**). The expression patterns of these markers in TCGA and GSE68465 were presented in **Figures 1C, D**. Using these CAFs markers, the infiltration of CAFs in LUAD was quantified (**Figures 1E, F**). Based on the GSEA analysis, we explored the potential pathways in different CAFs infiltration groups. The results based on the GO gene set revealed that adaptive immune response, blood vessel morphogenesis and calcium ion transport are associated with high CAFs infiltration. In addition, results based on the Hallmark gene set showed that the EMT, inflammatory response, and IL6/JAK/STAT3 are activated in patients with high CAFs infiltration (**Figures 2A, B**). The TIDE score was used to evaluate tumor immune escape mechanisms. We found that CAFs infiltration was positively correlated with the TIDE score (**Figure 2C**). Also, the LUAD patients with high CAF infiltration had a higher TIDE score, suggesting a worse immunotherapy response (**Figures 2D, E**).

### Identification of the CAFs characteristics molecules based on the machine learning algorithm

**Figure 3:**
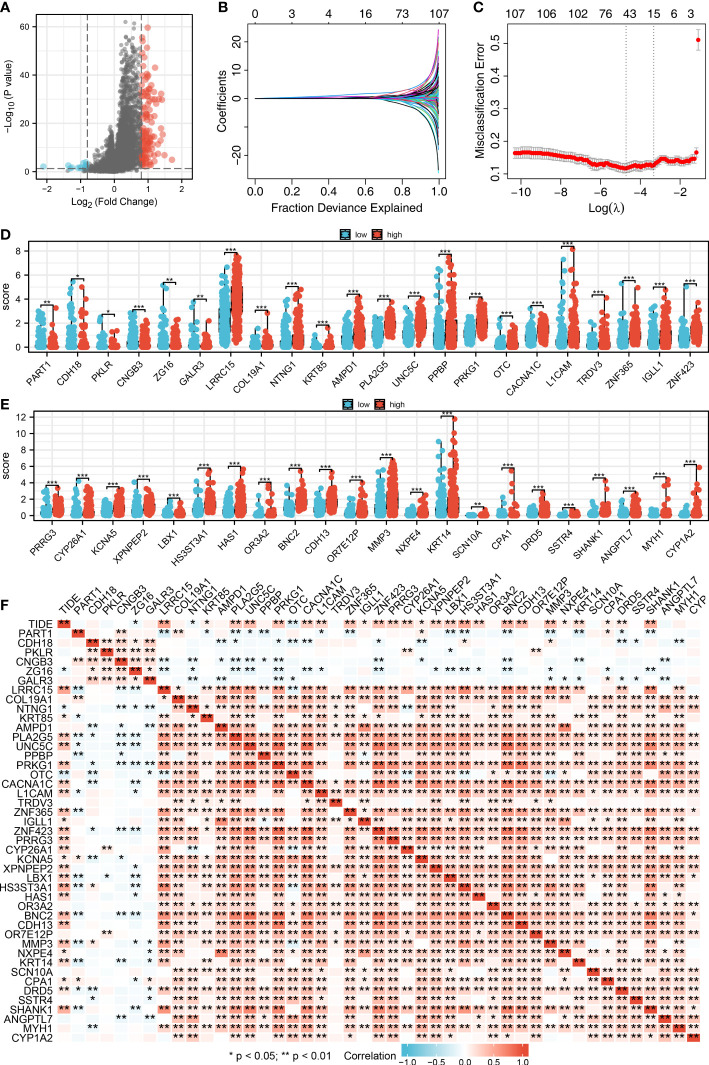
Identification of characteristic molecules of lung cancer immunotherapy. **(A)** The volcano map demonstrated the differential expressed genes between high- and low-CAFs groups; **(B-C)** Logistic lasso regression; **(D-E)** The expression level 44 CAF characteristic genes in patients with high- and low-CAFs infiltration, *P < 0.05, **P < 0.01, ***P < 0.001; **(F)** The correlation analysis between 44 CAF-related signatures and TIDE score, *P < 0.05, **P < 0.01.

We then performed the differential expressed analysis between the patients with high- and low-CAFs infiltration (**Figure 3A**). Subsequently, the LASSO logistics regression was used to identify the characteristic molecules of CAFs with the best optimization (**Figures 3B, C**). A total of 44 characteristics molecules were identified when the misclassification error is the lowest. Additionally, we found that the 44 characteristic molecules were closely associated with the TIDE score, which suggests that 44 characteristics molecules may be essential in LUAD immunotherapy (**Figures 3D, E**). The polygenic correlation plot also revealed the strong correlations between 44 characteristic molecules (**Figure 3F**).

### Establishment of a prognosis signature consisting of seven CAFs characteristics molecules

**Figure 4:**
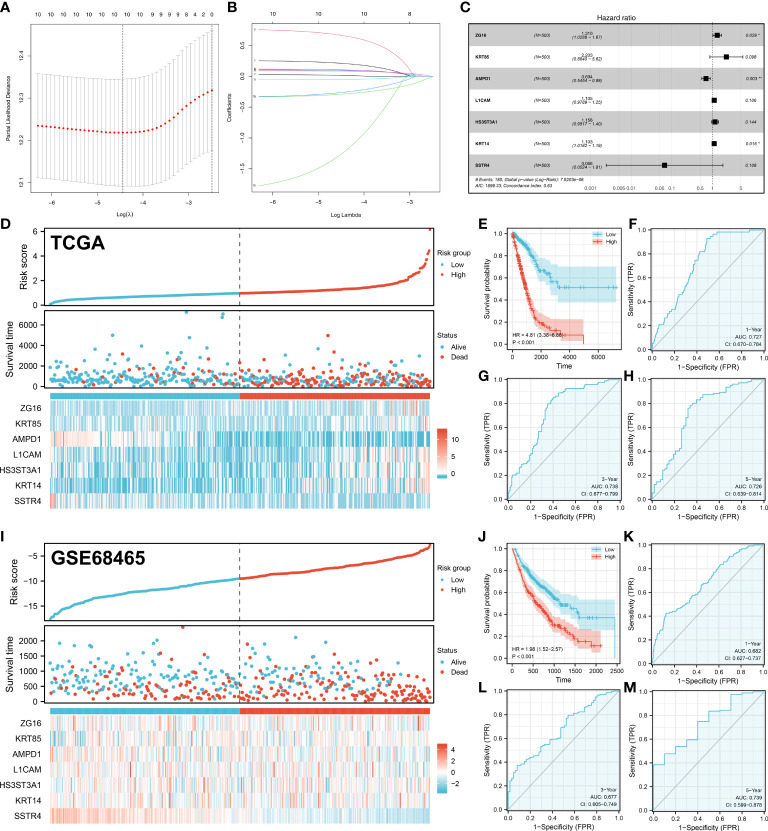
Prognosis model construction. **(A-B)** The lasso regression analysis was used for data dimension reduction; **(C)** A prognosis signature consisting of characteristic molecules was constructed by multivariate cox regression analysis; **(D)** LUAD patients involved in TCGA cohort was divided into low-risk and high-risk groups based on the median cut-off; **(E)** The Kaplan-Meier curve was used to demonstrate the OS between low-risk and high-risk groups in TCGA cohort; **(F)** The time-dependent ROC curves reveals the 1-year predictive value of prognostic in TCGA cohort; **(G)** The time-dependent ROC curves reveals the 3-year predictive value of prognostic in TCGA cohort; **(H)** The time-dependent ROC curves reveals the 5-year predictive value of prognostic in TCGA cohort; **(I)** LUAD patients involved in GEO cohort was divided into low-risk and high-risk groups based on the median cut-off; **(J)** The Kaplan-Meier curve was used to demonstrate the OS between low-risk and high-risk groups in GEO cohort; **(K)** The time-dependent ROC curves reveals the 1-year predictive value of prognostic in GEO cohort; **(L)** The time-dependent ROC curves reveals the 3-year predictive value of prognostic in GEO cohort; **(M)** The time-dependent ROC curves reveals the 5-year predictive value of prognostic in GEO cohort;. *P < 0.05, **P < 0.01.

Based on 44 characteristic molecules identified by the machine learning algorithm, we performed univariate cox regression, LASSO regression, and multivariate cox regression for prognosis model establishment (**Figures 4A-C**). Ultimately, seven characteristic molecules were involved in the prognosis model, including ZG16, KRT85, AMPD1, L1CAM, HS3ST3A1, KRT14 and SSTR4. Each LUAD patient was assigned a risk score with the formula of “Risk score =ZG16 * 0.270 + KRT85 * 0.789 + AMPD1 * -0.366 + L1CAM * 0.099 + HS3ST3A1 * 0.145 + KRT14 * 0.098 + SSTR4 * -2.716. According to the median cut-off, all the LUAD patients were divided into different risk groups based on risk score (**Figures 4D, I**). In TCGA and GSE68465 cohorts, according to KM survival analysis, the LUAD patients with lower risk score had better overall survival (OS) (**Figures 4E, J**). As illustrated by the time-dependent ROC curve, our model showed a satisfactory prediction performance (1-year accuracy is 0.727, 3-year accuracy is 0.738, 5-year accuracy is 0.726) in the TCGA cohort (**Figures 4F-H**). For GEO cohort, similar results were observed (1-year accuracy = 0.682, 3-year accuracy = 0.677, 5-year accuracy = 0.739) (**Figures 4K-M**).

### Clinical correlation of the model and seven characteristics molecules

**Figure 5:**
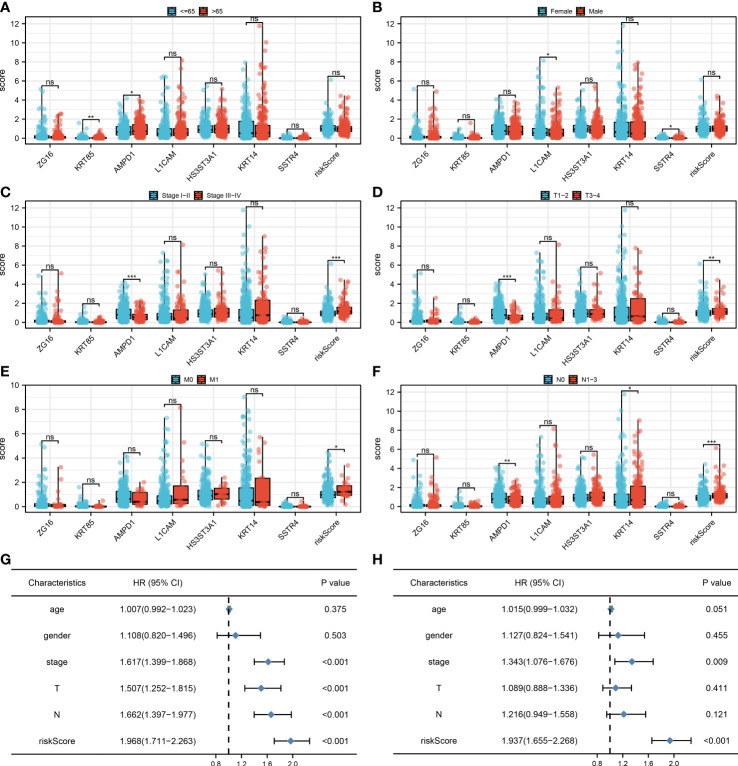
Clinical correlation. **(A)** The correlation analysis between age and expression level of seven characteristic molecules, ns P > 0.05, *P < 0.05, **P < 0.01; **(B)** The correlation analysis between gender and expression level of seven characteristic molecules, ns P > 0.05, *P < 0.05; **(C)** The correlation analysis between stage and expression level of seven characteristic molecules, ns P > 0.05, ***P <0.001; **(D)** The correlation analysis between T stage and expression level of seven characteristic molecules, ns P > 0.05, **P < 0.01, ***P <0.001; **(E)** The correlation analysis between M stage and expression level of seven characteristic molecules, ns P > 0.05, *P < 0.05; **(F)** The correlation analysis between N stage and expression level of seven characteristic molecules, ns P > 0.05, *P < 0.05, **P < 0.01, ***P <0.001; **(G)** The univariate independent prognostic analysis revealed that stage, T stage, N stage and risk score are independent prognostic factors in LUAD patients; **(H)** The multivariate independent prognostic analysis reveals that stage and risk score are independent prognostic factors in LUAD patients.

We found that clinical features like the clinical stage, T, N, and M classifications are significantly influenced by the risk score. Additionally, AMPD1 expression levels are closely associated with many clinical characteristics, such as age, stage, T stage, and N stage (**Figures 5A-F**). Results of the univariate analysis revealed that risk score is an independent prognostic factor in LUAD patients. For multivariate analysis, the same conclusion was get (**Figures 5G, H**).

### Distribution of immune cell subsets and immunotherapy response

**Figure 6:**
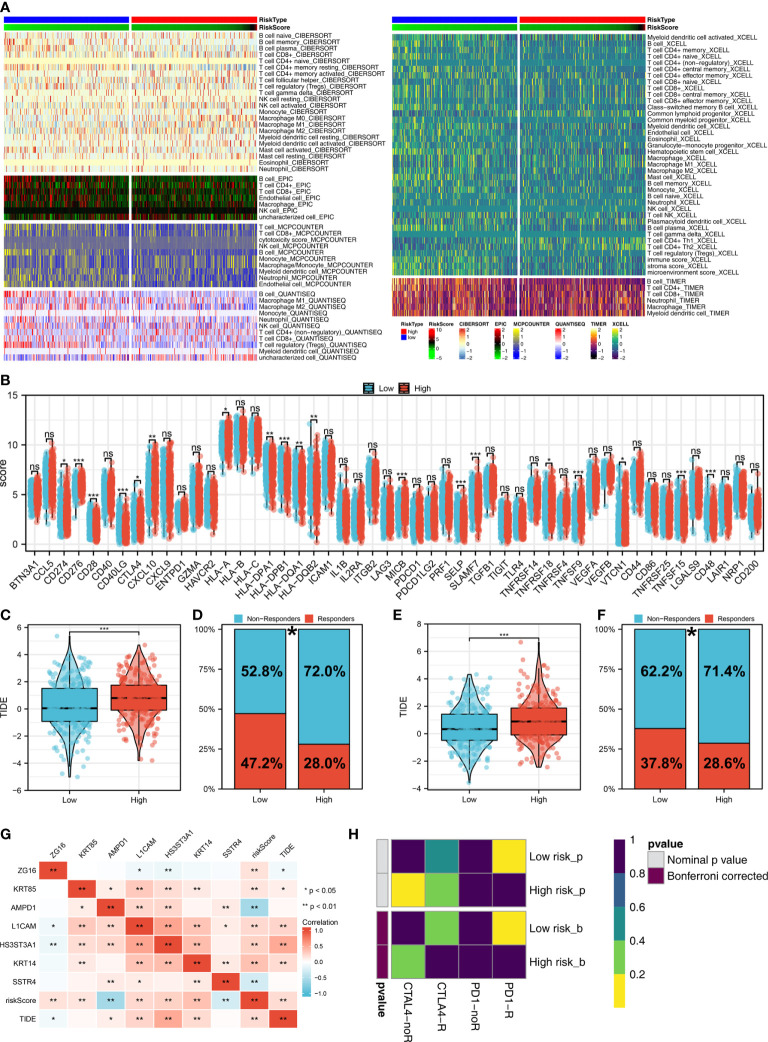
Immune-related analysis. **(A)** The correlation between risk score and various immune scores; **(B)** The expression level of immune-related genes in low- and high-risk groups, ns P > 0.05, *P < 0.05, **P < 0.01, ***P < 0.001; **(C)** The level of TIDE score in low- and high-risk groups in the TCGA cohort, ***P < 0.001; **(D)** Percentage of immunotherapy responders and non-responders in patients with high and low risk score (TCGA cohort), *P < 0.05; **(E)** T The level of TIDE score in low- and high-risk groups in the GSE68465 cohort, ***P < 0.001; **(F)** Percentage of immunotherapy responders and non-responders in patients with high and low risk score (GSE68465 cohort), *P < 0.05; **(G)** The correlation analysis between TIDE score, risk score and seven characteristic molecules, *P < 0.05, **P < 0.01; **(H)** The correlation analysis between risk score and the immunotherapy response of PD-1 and CTLA4.

**Figure 7:**
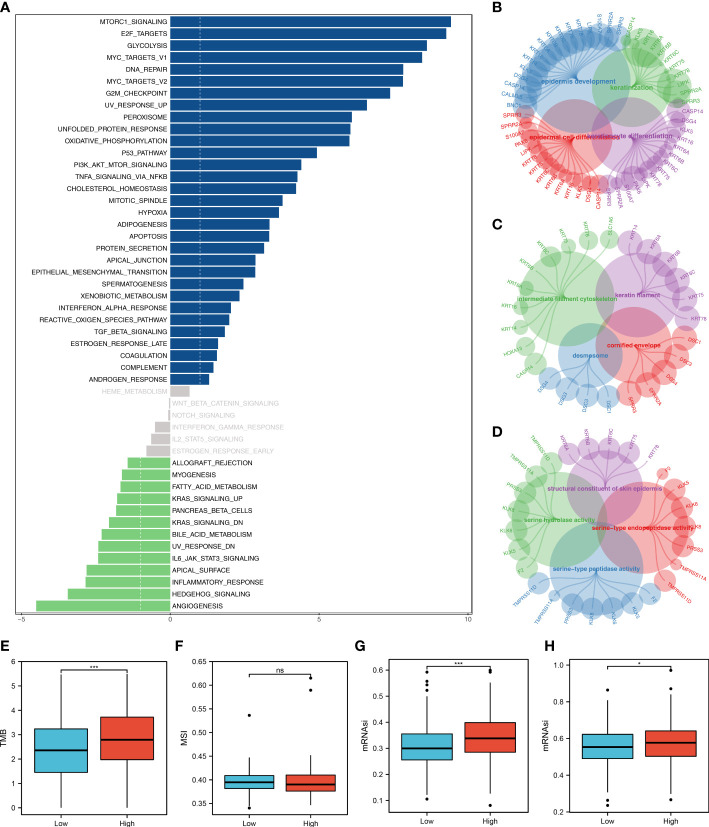
Biological enrichment analysis. **(A)** The GSVA analysis between low- and high-risk groups; **(B)** The GO-BP enrichment analysis of our model; **(C)** The GO-CC enrichment analysis of our model; **(D)** The GO-MF enrichment analysis of our model; **(E)** The boxplot demonstrated the TMB score in low- and high-risk groups, ***P < 0.001; **(F)** The boxplot demonstrated the MSI score in low- and high-risk groups, ns P > 0.05; **(G)** The boxplot demonstrated the mRNAsi score in low- and high-risk groups, ***P < 0.001; **(H)** The boxplot demonstrated the EGFR-mRNAsi score in low- and high-risk groups, *P < 0.05.

The tumor microenvironment contains many different cells, such as fibroblasts, immune cells, inflammatory mediators, and cancer cells with varying properties and chemical properties. Among them, immune cells are important in diagnosis, survival outcomes, and drug sensitivity in tumors. **Figure 6A** shows the relationship between risk score and various immune cells. Briefly, the risk score can remarkably affect the LUAD immune microenvironment. Subsequently, we examined the genes encoding human leukocyte antigens (HLA) and immune checkpoint blocking (ICB). Some reduction in immunological HLA gene and ICB-related gene expression was observed in the low-risk group, suggesting that tumor cells involved in the high-risk group evade immune surveillance more (**Figure 6B**). Also, in TCGA and GSE68465 cohorts, the LUAD patients in the high-risk group were correlated with higher TIDE scores, indicating a worse response to immunotherapy (**Figures 6C-F**). The correlation heatmap demonstrated significant associations between seven characteristics molecules, risk score and TIDE score (**Figure 6G**). In addition, the patients with lower risk score might respond better to the therapy of PD-1 (**Figure 6H**). Then, we explored the difference in the potential biological function in patients with high- and low-risk groups by GSVA and GO enrichment analysis. The GSVA enrichment analysis revealed that MTORC1 signaling, E2F targets, glycolysis and MYC target are the most enriched terms in the high-risk group, while angiogenesis, hedgehog signaling and inflammatory response are the most enriched terms in low-risk group (**Figure 7A**). For GO enrichment analysis, the enriched pathways involve biological process (BP), cell component (CC) and molecular function (MF). Keratinocyte differentiation, keratinization, and epidermal cell differentiation are the most enriched GO-BP pathways (**Figure 7B**). For GO-CC, cornified envelope and desmosome are the most enriched pathways (**Figure 7C**). The results of GO-MF revealed that structural constituents of skin epidermis, serine-type endopeptidase activity and serine hydrolase activity are the most relative pathways (**Figure 7D**). Tumor mutational burden (TMB) and microsatellite instability (MSI) are important biomarkers for immunotherapy response. We found that the patients in the high-risk group were associated with higher TMB, but not MSI (**Figure 7E, F**). The stemness index (SI) is also an indicator that determines whether tumor cells are similar to stem cells and correlates with tumor dedifferentiation. The results revealed that risk score were associated with higher EREG-mRNAsi and mRNAsi (**Figures 7G, H**).

### Role of AMPD1 in LUAD and its correlation with important immune checkpoints

**Figure 8:**
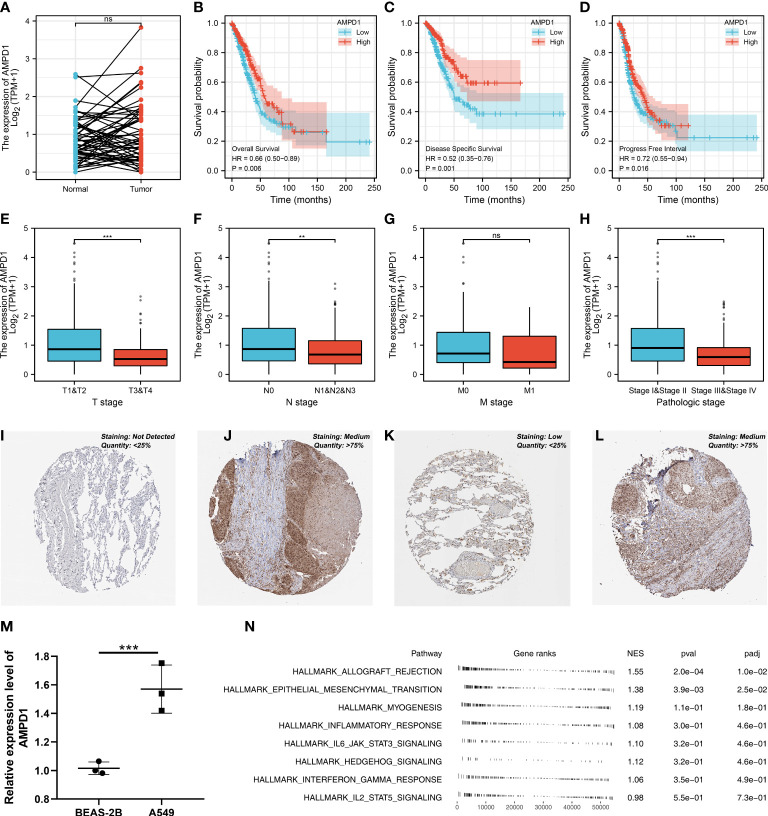
Further exploration of AMPD1 in lung cancer. **(A)** The expression level of AMPD1 in paired LUAD and control tissue, ns P > 0.05; **(B)** The OS in patients with low and high AMPD1 expression level; **(C)** The DSS in patients with low and high AMPD1 expression level; **(D)** The PFI in patients with low and high AMPD1 expression level; **(E)** The correlation analysis between expression level of AMPD1 and T stage, ***P <0.001; **(F)** The correlation analysis between expression level of AMPD1 and N stage, **P < 0.01; **(G)** The correlation analysis between expression level of AMPD1 and M stage, ns P > 0.05; **(H)** The correlation analysis between expression level of AMPD1 and stage, ***P < 0.001; **(I)** The AMPD1 protein level in normal lung tissue; **(J)** The AMPD1 protein level in LUAD tissue; **(K)** The AMPD1 protein level in normal lung tissue; **(L)** The AMPD1 protein level in LUAD tissue; **(M)** The qRT-PCR assay demonstrated the expression level of AMPD1 in A549 and BEAS-2B cells, ***P < 0.001; **(N)** The GSEA analysis of AMPD1.

**Figure 9:**
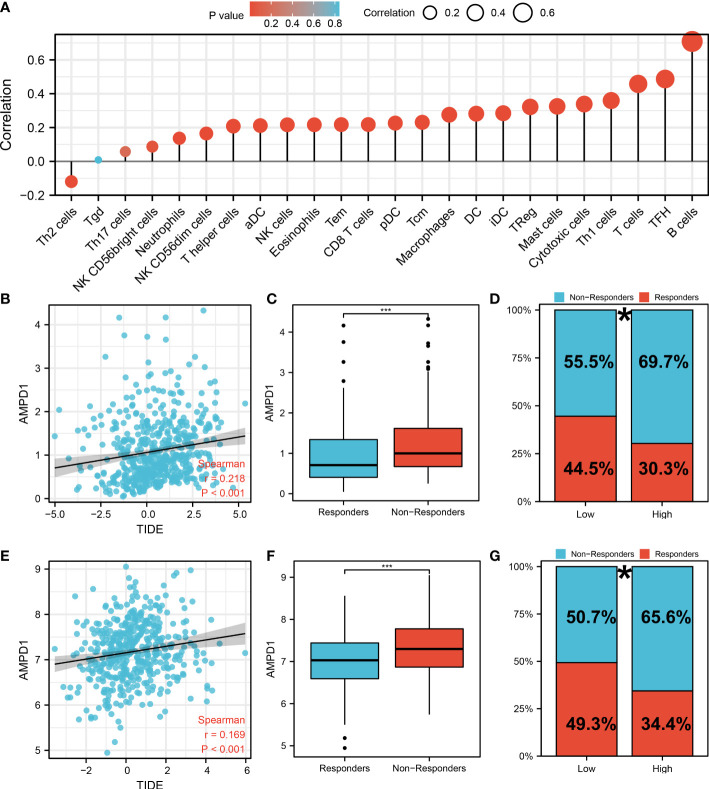
Immune-related analysis of AMPD1 in lung cancer. **(A)** The immune cell infiltration reveals that B cells, TFH, T cells, Th1 cells, cytotoxic cells and mast cells are positively correlated with the expression of AMPD1; **(B)** The correlation analysis between TIDE score and expression level of AMPD1 in TCGA cohort; **(C)** The AMPD1 expression in immunotherapy responders and non-responders (TCGA cohort), ***P < 0.001; **(D)** Percentage of immunotherapy responders and non-responders in high- and low-risk patients (TCGA cohort), *P <0.05; **(E)** The correlation analysis between TIDE score and expression level of AMPD1 in GSE68465 cohort; **(F)** The AMPD1 expression in immunotherapy responders and non-responders (GSE68465 cohort), ***P < 0.001; **(G)** Percentage of immunotherapy responders and non-responders in high- and low-risk patients (GSE68465 cohort), *P < 0.05.

**Figure 10:**
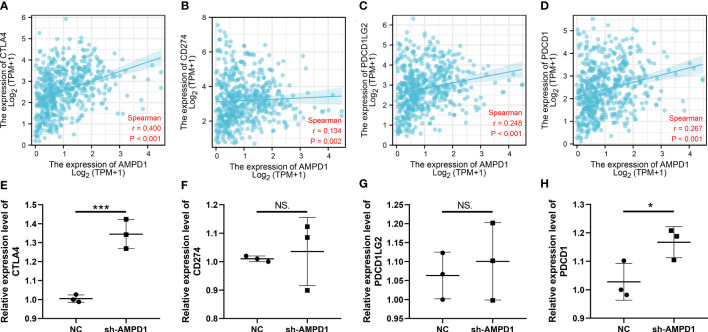
Correlation of AMPD1 and key immune checkpoints. **(A-D)** Correlation between AMPD1 and key immune checkpoints; **(E-H)** RNA level of key immune checkpoint in AMPD1 knockdown and control cells, NS P > 0.05, *P < 0.05, ***P < 0.001.

AMPD1 appears to play a crucial role in LUAD, according to the previous analysis. For a deeper understanding the role of AMPD1 in LUAD patients, we then performed differential expressed analysis, survival analysis, immunohistochemistry, PCR, GSEA, immune cell infiltration and TIDE score of AMPD1. No significant difference in AMPD1 mRNA level was found between the paired LUAD and the control tissue (**Figure 8A**). The survival analysis demonstrated that the patients with high AMPD1 levels might have better OS, disease-specific survival (DSS) and progress-free interval (PFI) (**Figures 8B-D**). In addition, the clinical analysis demonstrated that AMPD1 is closely associated with some clinical characteristics, such as clinical stage, T stage and N stage (**Figures 8E-H**). Immunohistochemical showed that the protein level of AMPD1 was higher in the LUAD tissue (**Figures 8I-L**). Also, the qRT-PCR result indicated that AMPD1 is upregulated in A549 cells compared with BEAS-2B cells at mRNA level (**Figure 8M**). The GSEA analysis revealed that allograft rejection, EMT, myogenesis, inflammatory response and IL6-JAK-STAT3 signaling may be the key pathways AMPD1 involved in (**Figure 8N**). The immune cell infiltration also revealed a positive correlation between AMPD1 expression and B cells, TFH, T cells, Th1 cells, cytotoxic cells, and mast cells (**Figure 9A**). Also, a positive correlation was observed between CAFs and AMPD1 (**Figure S2**). Further, in GEO and TCGA databases, we found that AMPD1 is associated with higher TIDE scores. LUAD patients with high AMPD1 expression might respond better to immunotherapy (**Figures 9B-G**). Considering the effect of AMPD1 on immunotherapy response, we next evaluated the correlation between AMPD1 and four key immune checkpoints, CD274, PDCD1, CTLA4 and PDCD1LG2. Results indicated that AMPD1 was positively correlated with these checkpoint molecules, especially CTLA4 (**Figures 10A-D**). The qRT-PCR result of cell lines showed that knockdown of AMPD4 can significantly reduce the expression of CTLA4 and PDCD1, but not CD274 and PDCD1LG2 (**Figures 10E-H**).

## Discussion

There are more than one million deaths worldwide each year caused by LUAD. Although new diagnostic and therapeutic technologies have been developed, it remains a serious global public health problem lacking effective advanced diagnostics and treatment options (25). Consequently, it is necessary to develop novel and effective markers to improve the early diagnosis and treatment of LUAD.

Cancer progression is believed to be largely driven by CAFs, which may be valuable therapeutic targets (26). Several studies indicated that CAFs are closely correlated with the development and occurrence of lung cancer. According to previous studies, miR-210 released by CAFs-exosomes has a role in promoting migration and invasion in lung cancer cells (27). In addition, FUT8/CF in CAFs propagated aggressive and malignant tissue microenvironments, which led to the faster proliferation and greater invasiveness of lung cancer in vivo and in vitro (28). In this work, based on the ssGSEA algorithm and single-cell analysis, LUAD patients were divided into different risk groups. Meanwhile, the patients with different CAFs infiltration levels showed different response patterns to immunotherapy. Subsequently, after screening differential expressed genes, we finally constructed a prognostic prediction model based on seven CAFs characteristics molecules.

Researchers have found that CAFs can significantly affect the cancer microenvironment. By inducing immunosuppressive macrophages, CAF may lead to an immunosuppressive environment (29). In a study by Zhao et al., the infiltration of CAFs can induce regulatory T-Cell (Treg) infiltration and indicate poor prognosis of oral squamous cell carcinoma patients (30). Moreover, CAFs promote the accumulation of tumor-promoting macrophages, resulting in immunosuppression (31). In this study, we found that the risk score has closely correlated with many immune-related pathways and patients with lower risk score might respond better to immunotherapy. In addition, the reduction in immunological expression of HLA genes and immune checkpoint genes was higher in the low-risk group. More specifically, the LUAD patients in the low-risk group show better outcomes when receiving immunotherapy of PD-1. Also, the biological enrichment revealed that CAFs was significantly correlated with immune response. In this work, we also discovered that AMPD1 was associated with many immune cells, including B cells and T cells. Moreover, we found that LUAD patients with a high expression level of AMPD1 are associated with higher TIDE scores. Accordingly, lung cancer patients who express high levels of AMPD1 have a worse response to immunotherapy.

In seven characteristics molecules involved in the prognosis prediction model, we found that AMPD1 is highly associated with clinical characteristics, such as age, clinical stage, T stage and N stage. Further, we found that the LUAD patients with higher AMPD1 expression levels were associated with better OS, DSS and PFI. The qRT-PCR assay demonstrated that the expression level of AMPD1 in A549 cells is higher than in normal lung cells. Immunohistochemistry showed that the protein expression level is inconsistent with the RNA level. According to numerous studies, many tumors are related to AMPD1. In breast cancer, a study showed that AMPD1 expression was closely linked to tumor-infiltrating immune cells and prognosis outcomes in HER2-positive breast cancer and that it may serve as a potential biomarker (32). In thyroid cancer, AMPD1 expression closely correlates with malignant evolution and the clinical prognosis of patients, and it is promising to become an important biomarker for immunotherapy (33).

Considering recent exciting developments in immunotherapy in cancer, it is meaningful to develop new and appealing immunotherapeutic approaches by utilizing innate immune cells. CAFs can influence the microenvironment of tumors. Hence, therapeutic strategies that reactivate the immunosuppressive microenvironment that CAF mediates may enhance conventional treatments as well as immunotherapies. Understanding the interaction between CAFs and tumor progression will allow new therapy clues to be identified. Meanwhile, some limitations should also be noticed. Firstly, the patients included in our study were primarily Western populations and the race bias might reduce the conclusion reliability. Secondly, the detailed information of patients was not clear, for instance, detailed laboratory inspection data, which might bring underlying bias.

## Data availability statement

Publicly available datasets were analyzed in this study. This data can be found here: https://portal.gdc.cancer.gov/, https://www.ncbi.nlm.nih.gov/gds/?term.

## Author contributions

WQ, WX, ZX and DK performed the analysis. WQ, WX and ZY performed the experiments and wrote the manuscript. CD and ZL designed this work. All authors participated in the article.

## Conflict of interest

The authors declare that the research was conducted in the absence of any commercial or financial relationships that could be construed as a potential conflict of interest.

## Publisher’s note

All claims expressed in this article are solely those of the authors and do not necessarily represent those of their affiliated organizations, or those of the publisher, the editors and the reviewers. Any product that may be evaluated in this article, or claim that may be made by its manufacturer, is not guaranteed or endorsed by the publisher.

## Supplementary material

Supplementary Figure 1 The flow chart of the whole study.

Supplementary Figure 2 The correlation analysis between CAFs infiltration and expression level of AMPD1.
