# Supplementary figures and images for: Machine learning identifies characteristics molecules of cancer associated fibroblasts significantly correlated with the prognosis, immunotherapy response and immune microenvironment in lung adenocarcinoma
Source: Front Oncol. 2022 Nov 9;12:1059253. doi: 10.3389/fonc.2022.1059253 (PMC9682016; doi:10.3389/fonc.2022.1059253)

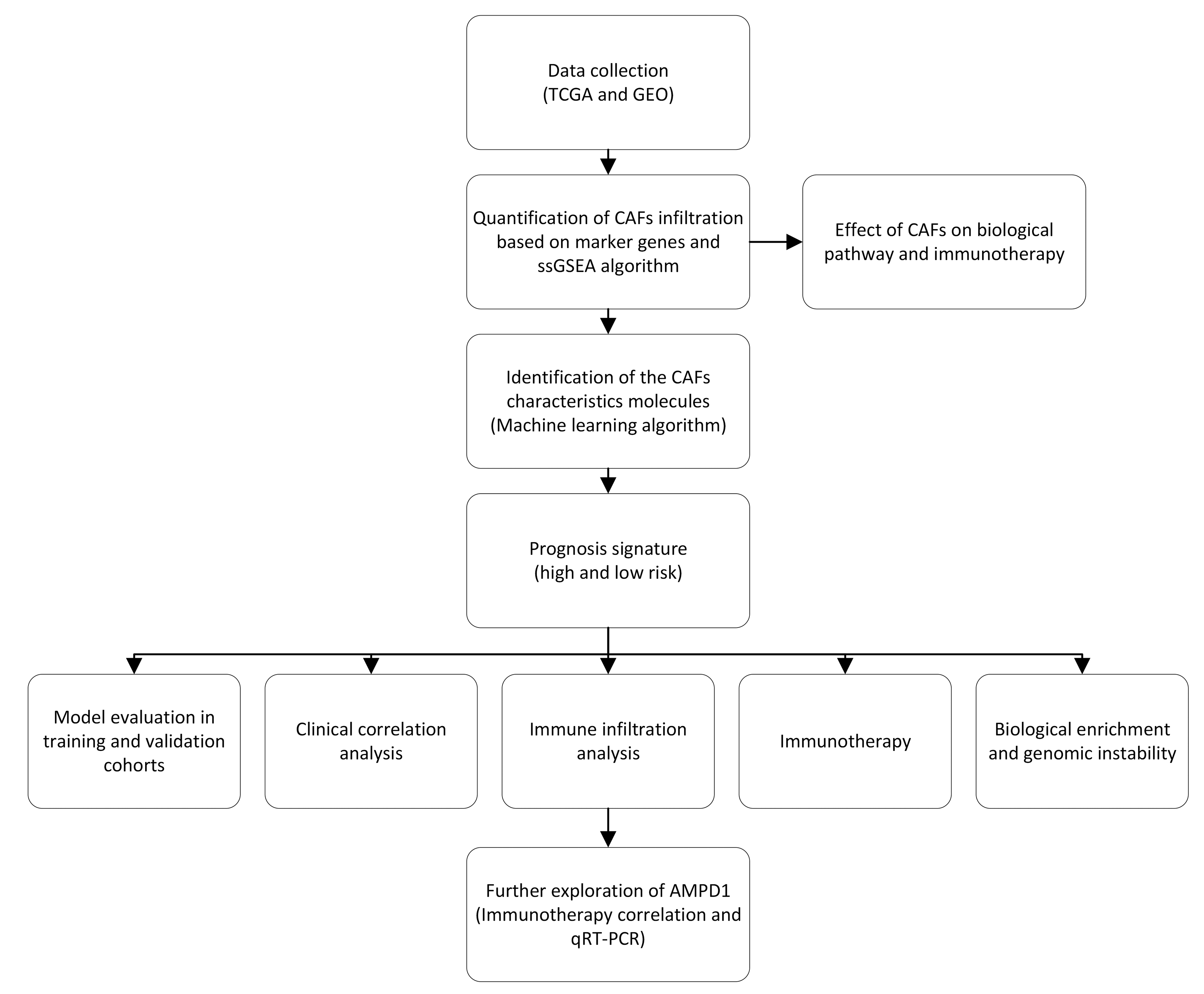

Supplement: Supplementary Figure 1 — The flow chart of the whole study. [file Image_1.tif]

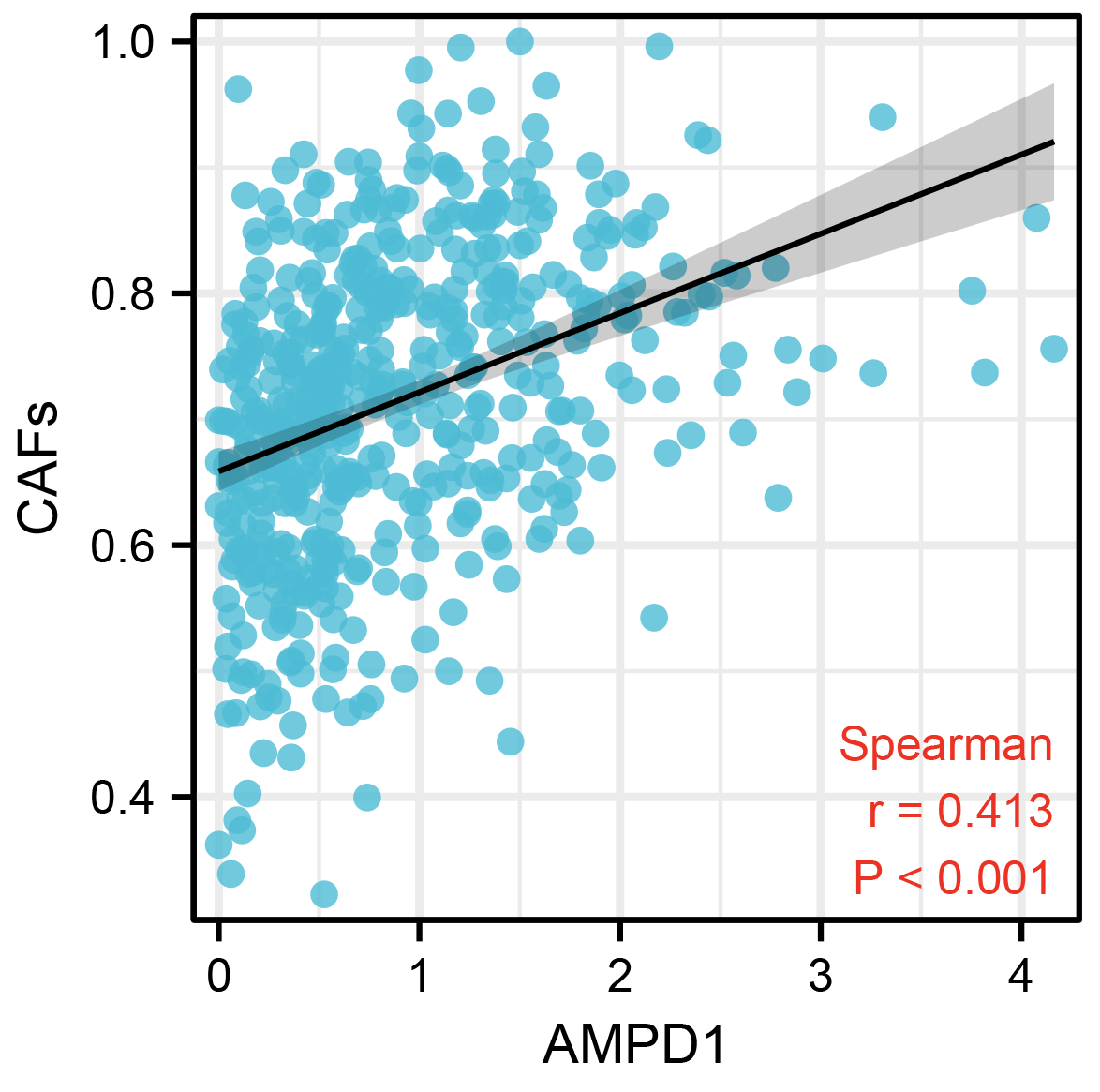

Supplement: Supplementary Figure 2 — The correlation analysis between CAFs infiltration and expression level of AMPD1. [file Image_2.tif]
